# Fasting increases investment in soma upon refeeding at the cost of gamete quality in zebrafish

**DOI:** 10.1098/rspb.2022.1556

**Published:** 2023-04-12

**Authors:** Edward R. Ivimey-Cook, David S. Murray, Jean-Charles de Coriolis, Nathan Edden, Simone Immler, Alexei A. Maklakov

**Affiliations:** ^1^ School of Biological Sciences, University of East Anglia, Norfolk NR4 7TJ, UK; ^2^ Institute of Biodiversity, Animal Health and Comparative Medicine, University of Glasgow, Glasgow, UK; ^3^ Collaborative Centre for Sustainable Use of the Seas (CCSUS), School of Environmental Sciences, University of East Anglia, Norfolk NR4 7TJ, UK; ^4^ The Centre for Environment, Fisheries and Aquaculture Science (Cefas), Lowestoft, Suffolk NR33 0HT, UK

**Keywords:** dietary restriction, fasting, soma, gamete, zebrafish, trade-off

## Abstract

Fasting increases lifespan in invertebrates, improves biomarkers of health in vertebrates and is increasingly proposed as a promising route to improve human health. Nevertheless, little is known about how fasted animals use resources upon refeeding, and how such decisions affect putative trade-offs between somatic growth and repair, reproduction and gamete quality. Such fasting-induced trade-offs are based on strong theoretical foundations and have been recently discovered in invertebrates, but the data on vertebrates are lacking. Here, we report that fasted female zebrafish, *Danio rerio*, increase investment in soma upon refeeding, but it comes at a cost of egg quality. Specifically, an increase in fin regrowth was accompanied by a reduction in 24 h post-fertilization offspring survival. Refed males showed a reduction in sperm velocity and impaired 24 h post-fertilization offspring survival. These findings underscore the necessity of considering the impact on reproduction when assessing evolutionary and biomedical implications of lifespan-extending treatments in females and males and call for careful evaluation of the effects of intermittent fasting on fertilization.

## 1. Introduction

In the past two decades, there has been a wealth of research highlighting the robust lifespan- and healthspan-extending effects of dietary restriction (DR) [1–3], defined as a reduction in nutrient intake without malnutrition. Much of this work has been directed towards the trade-off between reproduction and survival that typically occurs as part of an organism's DR response. Moreover, there are the two predominant evolutionary theories that seek to explain why this lifespan extension occurs [4,5]. Firstly, the resource allocation theory [5] suggests that organisms should reallocate resources away from reproduction into somatic maintenance and repair during periods of food limitation. In doing so, there is a negative effect on reproduction but an increase in the likelihood of surviving until food becomes plentiful again [5]. A more recent theory suggests that this lifespan extension is merely a by-product of upregulating autophagy and the breakdown of internal resources (in addition to a general laboratory artefact of reduced selection pressures) in order to maximize fitness in dietary restricted environments [4]. However, regardless of these two theories, which differ in the ultimate and proximate reasons for DR-mediated lifespan extension, there is a general observation across multiple taxa that increased survival during periods of transient fasting is often associated with reduced reproduction.

More recently, the adaptive nature of the DR response has come under scrutiny [6,7], particularly when individuals are brought back into an ad libitum environment. Whereas reduced reproduction during fasting could be adaptive when resources are limited, reducing the likelihood of producing offspring into a resource-poor environment and increasing the likelihood of surviving until the environment becomes resource-rich again, there is uncertainty about the effects of DR on survival and reproduction upon refeeding. Indeed, recent work in *Drosophila melanogaster* has shown that upon exiting a period of DR, not only is there an increase in mortality [6–8] but, under some circumstances, there is no compensation for this survival decline with increased fecundity, suggesting an overall cost of the DR response [5]. However, more recent work by Sultanova *et al*. [7] has shown a contrasting effect, with increased fecundity and mating behaviour in post-DR flies that suffered a similar increase in mortality [6]. There is, therefore, a distinct lack of consensus on whether the DR response is associated with increased reproduction and fitness upon refeeding, but there is also a lack of data on vertebrate species that extends beyond measuring the physiological effects of refeeding, including weight gain and quantity of adipose tissue ([8,9] although see [10]).

Another important factor to consider when determining the fitness effects of DR is the inter- (from parent-to-offspring) or trans-generational (from parents-to-grand-offspring and onwards) effects manifesting on future generations. In particular, recent studies in *Caenorhabditis elegans* and *Caenorhabditis remanei* [11–15] have revealed the significant impact that parental fasting can have on various measures of offspring performance, from mediating egg size and number [11] to influencing future survival in both immediate and distant generations [12]. Nevertheless, the effects of DR on fitness of offspring produced after parents resumed their normal diet is poorly understood and effects could be reversed if post-DR parents were in a better physiological condition. For instance, in a natural population of female Brandt's vole (*Lasiopodomys brandtii*), refeeding after a period of 70% food restriction caused survival rate of weaning offspring to increase by approximately 10%, suggesting that individuals were able to overcompensate for the reduced reproduction during the dietary restricted period [16]. However, it has been widely recognized that both maternal (egg- or care-mediated) and paternal (sperm- or care-mediated) phenotypes can readily and separately contribute to changes in offspring condition [17–25]. Thereby, not only does this warrant more research into other vertebrate taxonomic groups, but also the effects of refeeding should be discussed considering both maternal and paternal effects. This is especially relevant as the effects of DR have been found to differ markedly depending on the sex of the experimental individual (see [3]).

To this end, we investigated the effects of DR and subsequent refeeding on various important life-history characteristics in the model vertebrate system the zebrafish (*Danio rerio*)*.* We focused on addressing three key questions: (i) how do individuals allocate resources upon refeeding between somatic maintenance, fecundity, and offspring quality? (ii) do these responses differ between females and males? and (iii) do periods of fasting and refeeding have differing effects on offspring life-history traits such as survival and growth? We found that female zebrafish invest in their somatic maintenance upon refeeding and are better able to deal with somatic injury compared with control counterparts. However, this benefit comes at a cost of lower egg quality. Furthermore, we found that DR reduces quality of male gametes when males resume their normal feeding. Our findings highlight the importance of considering all aspects of an individual's life history, from fertilization and gametes to somatic maintenance and repair, in order to fully understand the implications of transient DR.

## 2. Methods

### (a) Animal husbandry and diets

All experimental assays used zebrafish (*D. rerio*) obtained from a population of outbred wild-type (AB strain) that originated from the European Zebrafish Research Centre (EZRC, Tübingen, Germany), raised to sexual maturity (more than or equal to 6 months old) and were maintained for up to two generations following an outbreeding regime at the controlled ecology facility (CEF) at the University of East Anglia (UEA, UK). Individual fish were housed in 3 l tanks in a recirculating rack system (ZebTec Active Blue Techniplast UK) at 26.4 ± 1.4°C and kept on a 12L : 12D light cycle. When fed ad libitum and prior to the experimental assays, they were fed two or three times a day with a mixture of live *Artemia* (brine shrimp cysts, approx. 100 *Artemia* per ml of water) and dry food (400–600, Sparos Zebrafeed) and kept at 1 : 1 sex ratios. Reproductively mature individuals of a standardized age (between 12 and 18 months) were then placed at random on one of two diets for 15 days, either fully-fed (ad libitum) or fasted (no food). After 15 days, the fasted individuals were placed back onto ad libitum food. Importantly, as egg eating occasionally occurs in this species, there is a risk of individuals not undergoing caloric restriction on the fasting diet. However, even if this behaviour did occur, the average weight loss observed in the fasted treatment (approx. 20%) suggested that consumption of eggs had little to no impact. Lastly, as zebrafish can display social dominance traits, leading to aggression against subordinate individuals, artificial aquaria plants were added for sheltering and hiding space to prevent aggressive interactions from influencing results within the current study [26].

Experimental protocols were approved by the Named Animal Care and Welfare Officer (NACWO) (project licence number: P0C37E901).

### (b) Caudal fin assay

To measure somatic growth, on day 3 of the experimental treatment a section (greater than or equal to 50%) of the caudal fin of 24 fed and 36 fasted fish (12 fed and 18 fasted males and females spread over five replicate blocks) was removed. The caudal fin, beginning from the caudal peduncle to the tip of each tail lobe, was then photographed including a scale bar. The surface area was then measured using ImageJ software [27] before and immediately after removal, and then on days 7, 15, 21, 28 and 35.

### (c) Reproductive assay

To test various measures of reproductive performance, 30 experimental males and 30 experimental females (again, with 12 fed and 18 fasted males and females) were paired on days 7, 15, 21, 28 and 35 of the experiment with wild-type fish of the opposite sex (which were again of a standardized age between 12 and 18 months and chosen at random) from the general stock population and placed in a breeding tank separated by a divider to allow for odour acclimatization (1 l Breeding Tanks, Techniplast). On the morning of breeding, the divider was removed and pairs were checked every hour to monitor spawning success. Fish were given a maximum of 5 h to spawn, after which, if they were unsuccessful, they were given another wild-type partner and the process was repeated the next day. Upon spawning, eggs were transferred into a Petri dish with tank water and methylene blue (0.1%) to prevent fungal infection. The eggs were then assessed after 2 and 24 h. Eggs were compared with embryonic diagrams produced by Kimmel *et al*. [28], which describe stages of zebrafish embryonic development. Eggs that deviated from the diagrams were classified as abnormal, and were classified as dead if they did not contain a yolk or if they were dark green or black in colour, or unfertilized if there was no cell division within the egg 2 h after sperm had been added [28]. Lastly, eggs were taken from day 5 of reproduction and used in the subsequent growth assay. Here, fry length, defined as the total length from the tip of the head to the end of the caudal fin, was measured at 3 and 5 days post-fertilization, again using ImageJ software [27]. In total, fry from 526 fed and 400 fasted individuals were measured (N.B. the total number was higher, but some were omitted as they were deformed or the eggs did not hatch; in addition, some individuals (approx. 124) only provided estimates of growth on 1 day, and were omitted from the additional analysis of total growth).

### (d) Sperm assay

Ejaculates were collected from each experimental male (12 for fasted and 12 for control fed) on days 7, 15, 21 and 35 of the experiment. To do this, all experimental fish were transferred to 1 l breeding boxes that contained an internally perforated tank. Fish from the same tank were kept together and were kept in absolute darkness at 28°C for 17 h without food to avoid contamination of samples with faeces. The males were then anaesthetized in 10 mg l^−1^ AquaClam (Western Chemicals) solution for a maximum of 60 s, briefly rinsed in system water and placed ventral side up into a moist sponge under a stereomicroscope. A paper towel was used to dry the area immediately adjacent to the ventral pore to avoid sperm coming into contact with water and prematurely activating. Sperm was then released by gently pressing the belly and collected in a micro capillary tube (Sigma-Aldrich, P0674). Collected sperm was added to microtubes containing 20 µl premix solution, consisting of 1 : 9 Hanks' buffer to distilled water, and placed on ice. Each sample was analysed within 2 h of extraction using a computer-assisted sperm analysis (CASA) system. For each sample, 2 µl of ejaculate–premix was activated with 3 µl of tank water. Of this 5 µl total mix, 3 µl was transferred to a chambered Cytonix MicroTool B4 Slide. Sperm movement was recorded at 100× magnification using a 782 M monochrome CCD progressive camera and a UOPUB203i trinocular microscope. The parameter settings used during this assay with the ISAS v.1 software were as follows: 50 frames s^−1^ 50 frames used, 5–50 µm^2^ particle area. We primarily assess curvilinear velocity (VCL) and average path velocity (VAP) as these parameters are most relevant for external fertilization success in teleost fish [29–31].

### (e) Statistical analysis

All analyses were conducted using R v.4.1 [32]. Data were tidied and cleaned using the Tidyverse v.1.3.1 [33], hablar v.0.3.0 [34] and janitor v.2.1.0 [35] collection of packages. Linear mixed effect models were run using glmmTMB v.1.1.2.3 [36] and checked for zero-inflation, overdispersion and overall fit using DHARMa v.0.4.5 [37]. In the results, we display estimated marginal means (EMMs) from each model and associated pairwise comparisons calculated using emmeans/emtrends v.1.7.2 [38]. Model results were then visualized using the ggeffects v.1.1.1 package [39]. Lastly, overall significance of models involving interactions were tested using type 3 ANOVAs from the car v.3.0-12 package (where model contrasts in this case were set to ‘sums to zero’) [40]. Lastly, if non-significant interactions were identified, these were removed in order to reduce model complexity; no other model selection took place aside from this simplification.

For each trait, aside from VCL, VAP, and offspring survival, we ran two models. The first contained data from both males and females to test for differences in (parental)-sex-specific responses. The second (and third) were female- and male-specific models that looked more closely at the differences between ad libitum and fasting treatments. Lastly, for all traits aside from fry growth, the analysis was split into *during* and *post-*fasting (or refeeding) in order to observe any differences in life-history trade-offs between these two dietary events.

Models for all traits were fitted as follows. For fin regrowth and fry growth per day, dietary treatment (two-way factor: fed/fasted) was fitted in a Gaussian model and included an interaction with the linear form of day (and if analysing both sexes together, with a three-way interaction of sex; note that in all cases sex was referring to the experimental individual in the parental generation) and the random effect of fish ID nested within experimental replicate. For fin regrowth, a random slope of day was added to each model to fully account for possible pseudoreplication (see [41]). In cases where convergence under the default optimizer (Nelder–Mead) did not occur, a different optimizer (Broyden–Fletcher–Goldfarb–Shanno, BFGS) was used; however, in one case (post-fasting females) a change of optimizer still did not lead to convergence and the random slope was subsequently removed from one level of the nested random effect, experimental replicate. It is important to note that the fin regrowth trait is a relative measure rather than absolute as the variation in fish size between the two treatments was supposed to be random and equal; therefore, original size of fish was not a necessary covariate to include in the regrowth models. For average hourly fry growth, replicate was instead fitted as a fixed effect and the model was run without a random effect structure. Reproduction was measured in two distinct ways. Firstly, age-specific reproduction was fitted with a similar structure to above (either a three-way interaction of the linear form of time, with treatment, and sex, or if in the sex-specific model with simply a two-way interaction between treatment and time), albeit with a negative binomial error distribution (treatment interacting with day, random effects of fish ID nested within experimental replicate). Secondly, the total number of offspring produced in either the fasting/refeeding period was fitted in a negative binomial model (in both cases, a Poisson model was originally considered but rejected owing to poor fit and significant overdispersion) with simply treatment as the main fixed factor (aside from the combined sex model, which again included a two-way factor of sex) and a random intercept of experimental replicate. In both cases, if significant zero-inflation was detected in the residuals of model (identified using DHARMa), then an additional zero-inflated component was added to each model. Offspring survival was modelled as a binomial response (surviving = 1, dead = 0) where dietary treatment interacted with day and the same random effect structure as age-specific reproduction was used. Lastly, log-transformed sperm VCL and VAP were analysed in a Gaussian model with the same fixed effect structure (treatment : day); however, a two-level factor of replicate block (two-level factor) was also added. Only fish ID was included as a random effect.

## 3. Results

See electronic supplementary material, table S1 for a full overview of the sex-specific results of fasting and refeeding.

### (a) Effect of transient fasting on soma

**Figure 1.**
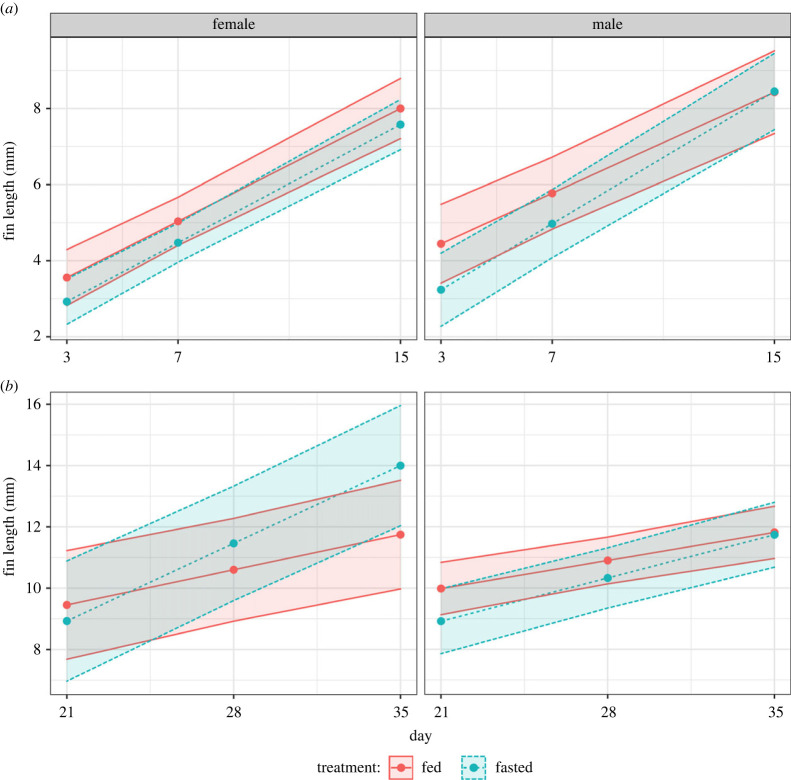
Sex-specific caudal fin regrowth prior to refeeding (*a*) and after refeeding (*b*). Colours represent fasted (blue, dashed lines) or fed (red, solid lines) dietary treatments in females (left) or males (right). Lines, points and 95% CIs represent predicted values from a linear model.

When assessing regrowth during the fasting period, there were no significant differences over time between males and females that were fasted or fed ($\chi_{\mathrm{df}=1}^{2}=1.64$ *p* = 0*.*200). Indeed, no detectable difference in regrowth rate was found between fasted and fed males (male fed: 0.36. male fasted: 0.42, estimate = −0.05 (0.051), *p* = 0.284; electronic supplementary material, table S2). A similar lack of difference was found in females (female fed: 0.40, female fasted: 0.37, estimate = 0.034 (0.05), *p* = 0.510; electronic supplementary material, table S2). Similarly, during the period of refeeding, males and females did not differ in regrowth rates between the fasted and fed treatments ($\chi_{\mathrm{df}=1}^{2}=0.48$, *p* = 0*.*487). Across both sexes, there was no detectable difference in the regrowth between fasted and fully-fed individuals (fed: 0.23, fasted: 0.25, estimate = −0.012 (0.03), *p* = 0.691; figure 1; electronic supplementary material, table S3). However, when looking at the sex-specific models, post-fasting females exhibited higher fin regrowth in comparison with those that were fed ad libitum (female fed: 0.16, female fasted: 0.36, estimate = −0.20 (0.07), *p* = 0.007; figure 1; electronic supplementary material, table S4; although note that in this particular case, an individual random slope was only fitted to the random effect of individual ID and not replicate).

### (b) Effect of transient fasting on offspring quality and quantity

**Figure 2.**
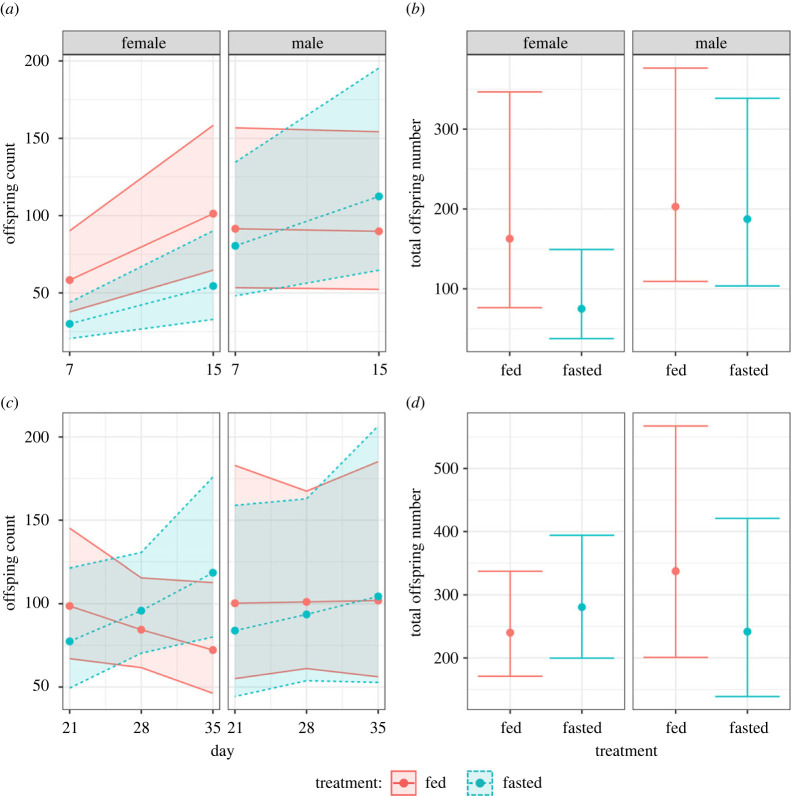
(*a*,*c*) Age-specific reproduction. Colours represent fasted (blue, dashed lines) or fed (red, solid lines) dietary treatments in females (left) or males (right). Lines, points and 95% CIs represent predicted values from a linear model. (*b*,*d*) Total number of offspring in either female (left, red) or males (right, blue). Lines, points and 95% CIs represent predicted values from a linear model. (*a*,*b*) Reproduction whilst individuals are within their respective dietary treatments. (*c*,*d*) Reproduction when all individuals have been placed back into ad libitum.

**Figure 3.**
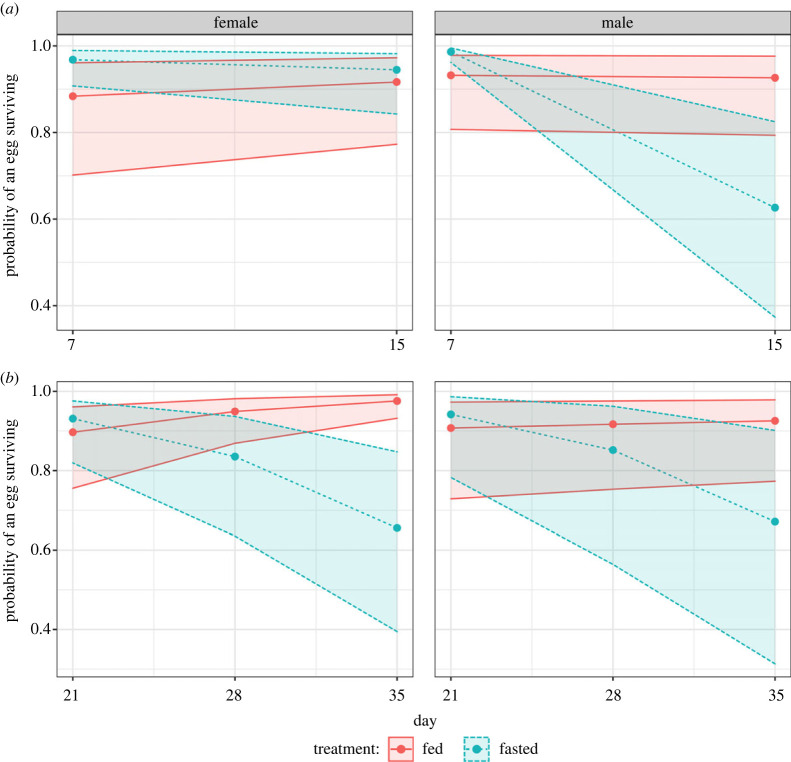
Age-specific egg survival during (*a*) or post-fasting (*b*). Colours represent fasted (blue, dashed lines) or fed (red, solid lines) dietary treatments in females (left) or males (right). Lines, points and 95% CIs represent predicted values from a linear model.

Transient fasting had sex-specific effects on the reproduction of males and females. When looking at both sexes combined, there were no differences in age-specific reproduction during the period of fasting ($\chi_{\mathrm{df}=1}^{2}=0.66,$ *p* = 0.416). Only in females was the average reproduction across both days 7 and 15 significantly lower in fasted individuals (EMM fed females: 89.5, fasted females: 46.6, contrast = 2.23, *p* = 0.0169; electronic supplementary material, table S5). This resulted in a reduced total number of offspring for females but not males during the dietary treatment period (total offspring: female fed = 163, female fasted = 75, estimate 2.56, *p* = 0.030; male fed = 203, male fasted = 187, estimate = 1.26, *p* = 0.726; electronic supplementary material, tables S6 and S7). This difference between fasted and fed individuals disappeared when females began to refeed (average reproduction EMM fed females: 92.7, fasted females: 105.0, contrast = 0.93, *p* = 0.566; electronic supplementary material, table S8a), resulting in a comparable total number of offspring between fed and fasted treatments (although we note the large increase in total number of offspring for females as they transition from fasting to refeeding; total offspring: fed = 240, fasted = 281, estimate = 0.856, *p* = 0.509; electronic supplementary material, table S8b). Male total offspring number on the other hand was unaffected by both the period of fasting (see above) and refeeding, with fasted individuals showing reduced reproduction (albeit not significantly so—total offspring EMM fed males: 338.0, fasted males: 242.0, estimate = 1.55, *p =* 0.162; electronic supplementary material, table S9). This reduced reproduction during fasting coincided with the relatively high egg survival of fasted females in comparison with fed females, although we note that fasted females still showed a significant decline (EMM fed females = 0.046, fasted females = −0.072, estimate = 0.12, *p =* 0.009; figure 2; electronic supplementary material, table S10). By contrast, males, despite not showing a detectable decline in offspring number, produced a large negative effect of fasting on the egg survival of their mate (EMM fed males = −0.01, fasted males = −0.47, estimate = 0.46, *p* < 0.001; figure 2; electronic supplementary material, table S11). Upon refeeding, the difference in trend between fasted and fed males became smaller, largely owing to a reduction in the negative effects of fasting; however, the effect size between the two treatments was still statistically different (EMM fed males = 0.017, fasted males = −0.15, estimate = 0.17, *p* < 0.001; figure 2; electronic supplementary material, table S12). For females, the opposite occurred, with fasted females seemingly trading off increased offspring number with a greater reduction in egg survival when fed ad libitum (EMM fed females = 0.11, fasted females = −0.14, estimate = 0.25, *p* < 0.001, denoted by the further decrease in slope value between fasting and refeeding periods; figure 3; electronic supplementary material, table S13).

### (c) Effect of transient fasting on sperm quality

**Figure 4.**
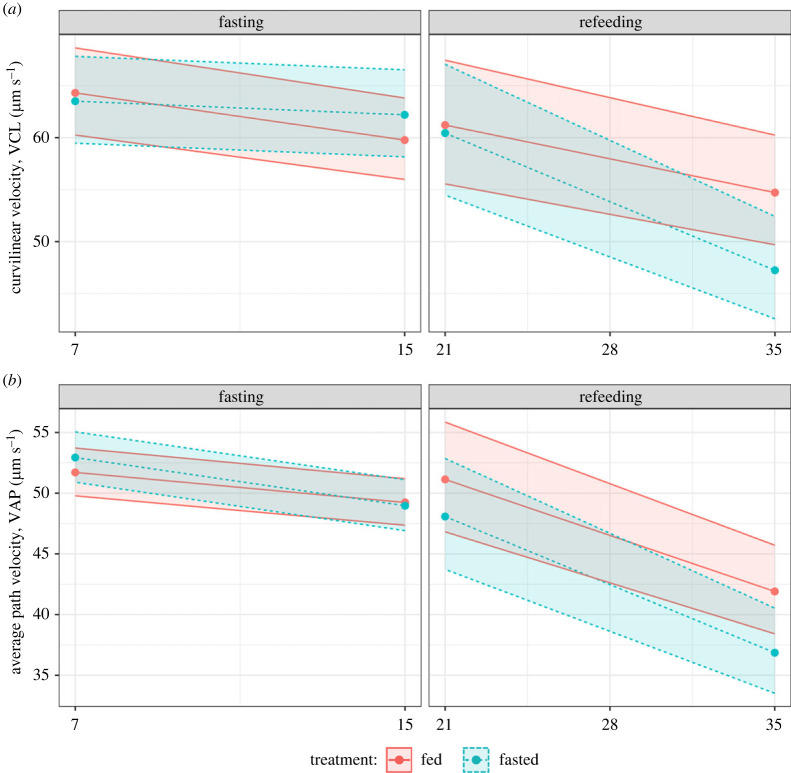
(*a*) Age-specific sperm curvilinear velocity (VCL) or (*b*) average path velocity (VAP) during (left) or post-fasting (right). Colours represent fasted (blue, dashed lines) or fed (red, solid lines) dietary treatments in males. Lines, points and 95% CIs represent predicted values from a linear model.

Male sperm quality, assessed through VCL and VAP, declined with time during both the fasting and refeeding periods (figure 4*a*,*b*). However, during the fasting period, the decline in VCL was greater for fed individuals (VCL EMM: fed = −0.009, fasted = −0.003, estimate = −0.007, *p <* 0.001; figure 4*a*; electronic supplementary material, table S14). By contrast, no detectable difference was found when observing VAP (VAP EMM: fed = −0.006, fasted = −0.01, estimate = −0.004, *p =* 0.062; figure 4*b*; electronic supplementary material, table S15). The opposite was true during the period of refeeding: the fasted individuals exhibited a faster decline in VCL with time (EMM: fed = −0.008, fasted = −0.018, estimate = 0.01, *p <* 0.001; figure 4*a*; electronic supplementary material, table S16); this was also consistent with the other measured sperm trait, VAP (EMM: fed = −0.014, fasted = −0.019, estimate = 0.05, *p <* 0.001; figure 4*b*; electronic supplementary material, table S17).

### (d) Effect of transient fasting on fry growth

**Figure 5.**
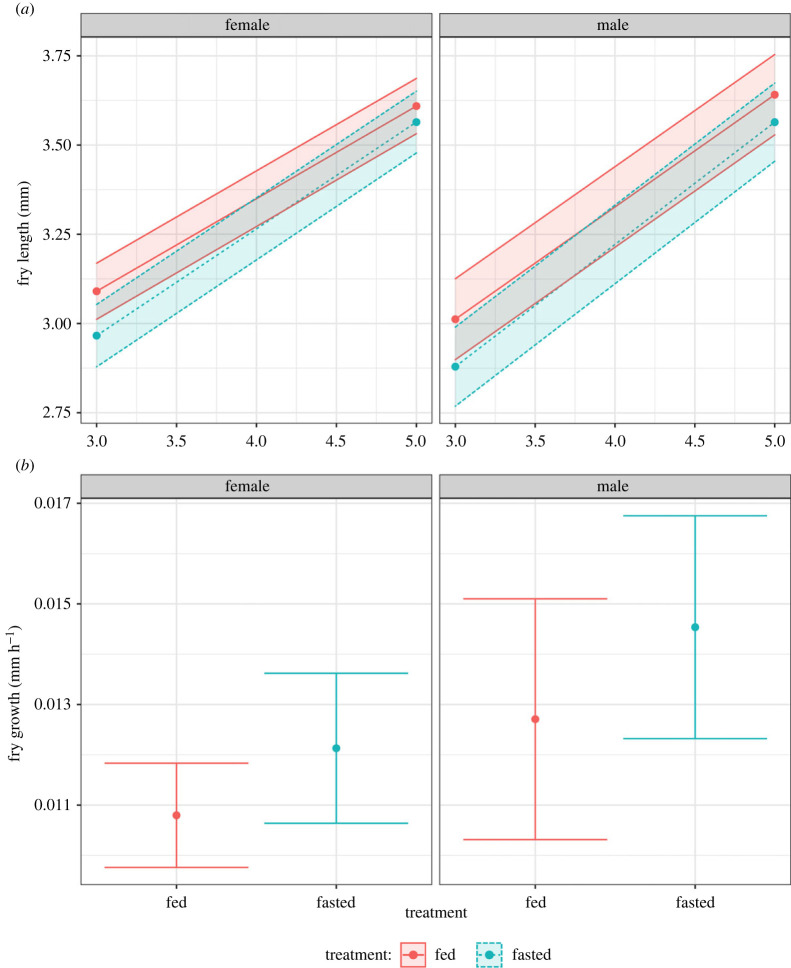
(*a*) Parental sex-specific fry growth between days 3 and 5 post-fertilization. Colours represent fasted (blue, dashed lines) or fed (red, solid lines) dietary treatments in female (left) or male parents (right). (*b*) Hourly fry growth in either female (left, red) or male parents (right, blue). Lines, points and 95% CIs represent predicted values from a linear model.

Sexes did not differ in average fry growth per day, with offspring from both fasted and fed males and females having similar growth rates ($\chi_{\mathrm{df}=1}^{2}=0.161,$ *p* = 0.689). However, across both treatments, offspring from males had faster average growth per day than offspring from females (growth rate EMM: females = 0.28, males = 0.33, estimate = −0.05, *p* < 0.001; figure 5; electronic supplementary material, table S18)*.* In a similar manner, across both sexes, offspring from fasted individuals grew faster than those from ad libitum parents (EMM: fed = 0.29, fasted = 0.32, estimate = −0.03, *p* = 0.025; figure 5; electronic supplementary material, table S18)*.* When looking within each sex, growth rates for offspring from fasted and fed males and females were not different from each other (EMM: fed males = 0.31, fasted males = 0.34, estimate = −0.03, *p* = 0.148; fed females = 0.26, fasted females = 0.30, estimate = −0.04 *p* = 0.074; figure 5; electronic supplementary material, tables S19 and 20). This difference, however, may have been driven in part by unequal sampling between time points on days 3 and 5. In the light of this, we refocused the analysis to investigate average growth rates per hour (figure 5) in order to obtain a higher resolution of analysis. Offspring from both males and females did not differ in their growth rates between the fed and fasted parental treatments ($\chi_{\mathrm{df}=1}^{2}=0.01,$ *p* = 0.911). However, across both parental treatments, offspring from male individuals had increased per hour growth rate (EMM: female = 0.012, male = 0.014, estimate = −0.002, *p* = 0.023; figure 5; electronic supplementary material, table S21)*.* Similarly, offspring from fasted individuals across both sexes had increased growth (EMM: fed = 0.012, fasted = 0.014, estimate = −0.002, *p* = 0.010; figure 5; electronic supplementary material, table S21). This difference was largely driven by offspring from male individuals, where in the sex-specific analysis only the difference between the hourly growth of the offspring from fed males and fasted males was significant (EMM: male fed = 0.013, male fasted = 0.015, estimate = −0.002, *p* = 0.028; female fed = 0.011, female fasted = 0.012, estimate = −0.001, *p* = 0.146; figure 5; electronic supplementary material, tables S22 and S23).

## 4. Discussion

During periods of DR, organisms increase investment into somatic maintenance and survival but lower investment into reproduction [5]. However, investment in reproduction can be subdivided into investment in offspring number and offspring quality (via germline maintenance and parental care) [42,43]. The putative trade-offs may comprise different combinations of traits [42,43]. While we detected no DR-related increase in investment into the soma when organisms were on low food, there was evidence of a trade-off between female fecundity, which was negatively affected, and offspring quality, which was maintained at a consistently high level (approx. 95%). Prioritizing offspring quality over quantity during periods of restricted food is a classic example of a commonly observed life-history trade-off [44–46]. Strikingly, this trade-off reversed upon refeeding in female zebrafish. Female reproduction increased, which compensated for the period of reduced nutrient intake, but it did so in conjunction with a reduction in offspring survival. A similar refeeding-related increase in reproduction following transient fasting was recently reported in *D. melanogaster* [7].

**Figure 6.**
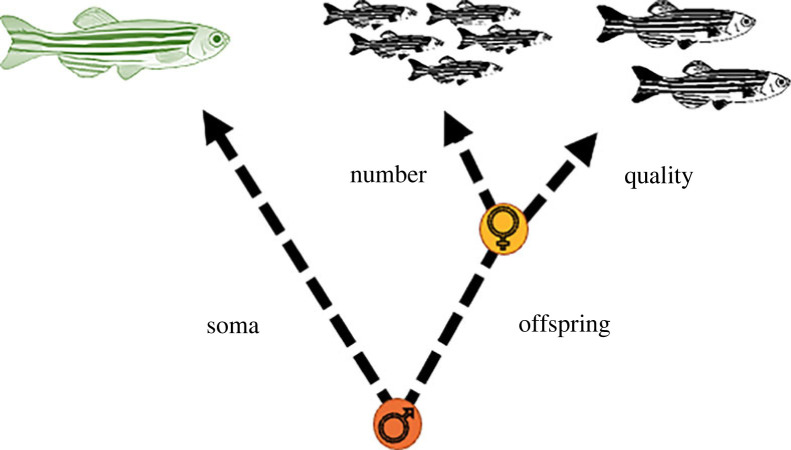
Proposed allocation decisions of females (yellow) and males (orange) after fasting when organisms resume full-feeding. We note that, for males, offspring traits are measured using a control female. Females trade offspring quality for offspring number and somatic maintenance. Males trade offspring number and quality for somatic maintenance. The orange or yellow filled circle denotes where the trade-off was observed during this experiment.

However, here we show that the increase in female reproduction is associated with a significant cost to gamete quality, resulting in reduced offspring survival. Furthermore, the increase in offspring number was associated with significant investment into the soma, represented by the increased fin regrowth rate upon refeeding. These results further suggest that trade-offs between somatic maintenance, offspring number and offspring quality are dynamic, and organisms change their allocation strategies depending on the environment. Specifically, post-DR trade-offs differ from what is typically observed, with females trading offspring quality for improved somatic maintenance and increased fecundity (figure 6). This suggests that while investment into germline production was occurring, there were not sufficient resources allocated into germline maintenance [42,43,47,48]. As a result, we could have expected an increase in germline mutations [43,47], which ultimately contributed to the reduced egg quality and egg survival during female refeeding. Importantly, owing to the average lifespan of these fish far exceeding the period of standardized age and dietary treatment, we can conclude that these results are due to some form of short-term trade-off and, therefore, not a product of senescent decline. Indeed, this effect may persist across multiple generations and produce not just inter- but also trans-generational effects of diet, as suggested in a recent study in *Drosophila* [49]. Furthermore, our findings may have direct implications for other scenarios of DR, such as intermittent fasting, which could be seen as a series of short fasting events. However, we note that a two-week fasting period in zebrafish cannot be directly compared with a two-week period in a mammal, owing to differences in metabolism.

A similar decline in gamete quality was found in males; however, our results indicated that while a reversible trade-off occurred between offspring quality and quantity in females, no such reverse occurred for males. This could in part be due to the fact that reproduction was measured using a control female. If females compensate for declining male quality, as suggested in a variety of different species [50,51], then reproductive number could be maintained at a high level regardless of the dietary environment of the male. We did, however, detect a significant decline in sperm quality, particularly when measuring sperm VCL, which was further exacerbated during the period of refeeding. This, coupled with the significant reduction in egg survival both during and after the fasting periods, suggests that regardless of this female compensation, males had traded-off gamete quality with investment into the soma (figure 6). This is in contrast to previous work in another species of fish, the guppy (*Poecilia reticulata*), which found no effect of dietary treatment on measured ejaculate traits [52–54], although we note that differences could instead manifest upon refeeding in this species. Indeed, it has been suggested that the relative amount of DR influences the probability of detecting diet-mediated changes to sperm traits. This could explain the lack of detectable effect observed in some studies, such as those listed previously, which contrasts with studies such as that here and [55].

Interestingly, offspring from fasted males exhibited significantly faster growth rate in comparison with those from ad libitum males. A similar trend was observed in offspring from the experimental females (albeit not statistically detectable), suggesting a form of compensatory growth. Such increased growth as a result of poor parental conditions has been found in another fish species, *Acanthochromis polyacanthus*. Here, juveniles from parents with low quality diets exhibited a sufficient increase in growth to catch up to the length of individuals from high quality diet parents (regardless of their own dietary environment) [25]. A similar effect was also found in three-spined sticklebacks (*Gasterosteus aculeatus*); however, in this particular study the effects were only investigated in the manipulated generation [56]. As a result of this adaptive increase in growth, we may expect costs to manifest in other traits, for instance in the strength of certain skeletal elements [57] or in a reduction in median lifespan [56].

Taken together, the detrimental effects of transient fasting manifested both during and, importantly, after fasting in both male and female gametes, in addition to the potential costs of compensatory offspring growth, suggest that future dietary interventions, particularly those aimed at healthspan extension in humans, should carefully evaluate the impacts of such regimes beyond the parental generation. Our findings show that transient fasting differentially affects gamete quality during and after fasting and that such effects depend on sex in a model vertebrate. Combined with previous work in invertebrate models, this research highlights the urgent need to investigate both short- and long-term effects of different forms of DR, and other interventions aimed at healthspan extension, on gamete quality and offspring health and fitness.

## Ethics

Experimental protocols were approved by the Named Animal Care and Welfare Officer (NACWO) (project licence number: P0C37E901).

## Data accessibility

R Code and data available from the Dryad Digital Repository: https://doi.org/10.5061/dryad.2280gb5w1 [58].

The data are also provided in the electronic supplementary material [59].

## References

[1] Fontana L, Partridge L. 2015 Promoting health and longevity through diet: from model organisms to humans. Cell **161**, 106-118. (10.1016/j.cell.2015.02.020) 25815989 PMC4547605

[2] Nakagawa S, Lagisz M, Hector KL, Spencer HG. 2012 Comparative and meta-analytic insights into life extension via dietary restriction. Aging Cell **11**, 401-409. (10.1111/j.1474-9726.2012.00798.x) 22268691

[3] Moatt JP, Nakagawa S, Lagisz M, Walling CA. 2016 The effect of dietary restriction on reproduction: a meta-analytic perspective. BMC Evol. Biol. **16**, 199. (10.1186/s12862-016-0768-z) 27717308 PMC5054627

[4] Adler MI, Bonduriansky R. 2014 Why do the well-fed appear to die young? A new evolutionary hypothesis for the effect of dietary restriction on lifespan. Bioessays **36**, 439-450. (10.1002/bies.201300165) 24609969

[5] Shanley DP, Kirkwood TBL. 2000 Calorie restriction and aging: a life-history analysis. Evolution **54**, 740-750. (10.1111/j.0014-3820.2000.tb00076.x) 10937249

[6] McCracken AW, Adams G, Hartshorne L, Tatar M, Simons MJP. 2020 The hidden costs of dietary restriction: implications for its evolutionary and mechanistic origins. Sci. Adv. **6**, eaay3047. (10.1126/sciadv.aay3047) 32128403 PMC7034997

[7] Sultanova Z, Ivimey-Cook ER, Chapman T, Maklakov AA. 2021 Fitness benefits of dietary restriction. Proc. R. Soc. B **8**, 20211787. (10.1098/rspb.2021.1787) PMC8611328 34814748

[8] Mair W, Goymer P, Pletcher SD, Partridge L. 2003 Demography of dietary restriction and death in *Drosophila*. Science **301**, 1731-1733. (10.1126/science.1086016) 14500985

[9] Kliewer KL, Ke J-Y, Stout MB, Cole R, Samuel VT, Shulman GI, Belury MA. 2015 Short-term food restriction followed by controlled refeeding promotes gorging behavior, enhances fat deposition, and diminishes insulin sensitivity in mice. J. Nutr. Biochem. **26**, 721-728. (10.1016/j.jnutbio.2015.01.010) 25913018 PMC4461460

[10] Boza JJ et al. 1999 Food deprivation and refeeding influence growth, nutrient retention and functional recovery of rats. J. Nutr. **129**, 1340-1346. (10.1093/jn/129.7.1340) 10395596

[11] Hibshman JD, Hung A, Baugh LR. 2016 Maternal diet and insulin-like signaling control intergenerational plasticity of progeny size and starvation resistance. PLoS Genet. **12**, e1006396. (10.1371/journal.pgen.1006396) 27783623 PMC5081166

[12] Ivimey-Cook ER, Sales K, Carlsson H, Immler S, Chapman T, Maklakov AA. 2021 Transgenerational fitness effects of lifespan extension by dietary restriction in *Caenorhabditis elegans*. Proc. R. Soc. B **288**, 20210701. (10.1098/rspb.2021.0701) PMC8113902 33975472

[13] Mautz BS, Lind MI, Maklakov AA. 2020 Dietary restriction improves fitness of aging parents but reduces fitness of their offspring in nematodes. J. Gerontol. A **75**, 843-848. (10.1093/gerona/glz276) PMC7164528 31761926

[14] Greer EL, Maures TJ, Ucar D, Hauswirth AG, Mancini E, Lim JP, Benayoun BA, Shi Y, Brunet A. 2011 Transgenerational epigenetic inheritance of longevity in *Caenorhabditis elegans*. Nature **479**, 365-371. (10.1038/nature10572) 22012258 PMC3368121

[15] Rechavi O, Houri-Ze'Evi L, Anava S, Goh WSS, Kerk SY, Hannon GJ, Hobert O. 2014 Starvation-induced transgenerational inheritance of small RNAs in *C. elegans*. Cell **158**, 277-287. (10.1016/j.cell.2014.06.020) 25018105 PMC4377509

[16] Xie X, Wen Y, Niu H, Shi D, Zhang Z. 2012 Re-feeding evokes reproductive overcompensation of food-restricted Brandt's voles. Physiol. Behav. **105**, 653-660. (10.1016/j.physbeh.2011.09.026) 22019786

[17] Watkins AJ. 2018 Paternal diet programs offspring health through sperm- and seminal plasma-specific pathways in mice. Proc. Natl Acad. Sci. USA **115**, 10 064-10 069. (10.1073/pnas.1806333115) PMC6176621 30150380

[18] Bonduriansky R, Head M. 2007 Maternal and paternal condition effects on offspring phenotype in *Telostylinus angusticollis* (Diptera: Neriidae). J. Evol. Biol. **20**, 2379-2388. (10.1111/j.1420-9101.2007.01419.x) 17956399

[19] Valtonen TM, Kangassalo K, Pölkki M, Rantala MJ. 2012 Transgenerational effects of parental larval diet on offspring development time, adult body size and pathogen resistance in *Drosophila melanogaster*. PLoS ONE **7**, e31611. (10.1371/journal.pone.0031611) 22359607 PMC3281084

[20] Preston BT, Saint Jalme M, Hingrat Y, Lacroix F, Sorci G. 2015 The sperm of aging male bustards retards their offspring's development. Nat. Commun. **6**, 6146. (10.1038/ncomms7146) 25647605 PMC4338826

[21] Kyneb A, Toft S. 2006 Effects of maternal diet quality on offspring performance in the rove beetle *Tachyporus hypnorum*. Ecol. Entomol. **31**, 322-330. (10.1111/j.1365-2311.2006.00775.x)

[22] Yanchula KZ, Alto BW. 2021 Paternal and maternal effects in a mosquito: a bridge for life history transition. J. Insect Physiol. **131**, 104243. (10.1016/j.jinsphys.2021.104243) 33845092

[23] Vuarin P, Lesobre L, Levêque G, Saint Jalme M, Lacroix F, Hingrat Y, Sorci G. 2021 Paternal age negatively affects sperm production of the progeny. Ecol. Lett. **24**, 719-727. (10.1111/ele.13696) 33565248

[24] Lardies MA, Carter MJ, Bozinovic F. 2004 Dietary effects on life history traits in a terrestrial isopod: the importance of evaluating maternal effects and trade-offs. Oecologia **138**, 387-395. (10.1007/s00442-003-1447-5) 14685846

[25] Donelson JM, Munday PL, McCormick MI. 2009 Parental effects on offspring life histories: when are they important? Biol. Lett. **5**, 262-265. (10.1098/rsbl.2008.0642) 19126532 PMC2665814

[26] Spence R, Gerlach G, Lawrence C, Smith C. 2008 The behaviour and ecology of the zebrafish, *Danio rerio*. Biol. Rev. Camb. Phil. Soc. **83**, 13-34. (10.1111/j.1469-185X.2007.00030.x) 18093234

[27] Abràmoff MD, Magalhães PJ, Ram SJ. 2004 Image processing with ImageJ. Biophotonics Int. **11**, 36-42.

[28] Kimmel CB, Ballard WW, Kimmel SR, Ullmann B, Schilling TF. 1995 Stages of embryonic development of the zebrafish. Dev. Dyn. **203**, 253-310. (10.1002/aja.1002030302) 8589427

[29] Zajitschek S, Hotzy C, Zajitschek F, Immler S. 2014 Short-term variation in sperm competition causes sperm-mediated epigenetic effects on early offspring performance in the zebrafish. Proc. R. Soc. B **281**, 20140422. (10.1098/rspb.2014.0422) PMC4024299 24789902

[30] Lahnsteiner F, Berger B, Weismann T, Patzner RA. 1998 Determination of semen quality of the rainbow trout, *Oncorhynchus mykiss*, by sperm motility, seminal plasma parameters, and spermatozoal metabolism. Aquaculture **163**, 163-181. (10.1016/S0044-8486(98)00243-9)

[31] Casselman SJ, Schulte-Hostedde AI, Montgomerie R. 2006 Sperm quality influences male fertilization success in walleye (*Sander vitreus*). Can. J. Fish. Aquat. Sci. **63**, 2119-2125. (10.1139/f06-108)

[32] R Core Team. 2021 R: a language and environment for statistical computing. Vienna, Austria: R Foundation for Statistical Computing. See https://www.R-project.org/.

[33] Wickham H et al. 2019 Welcome to the Tidyverse. *J. Open Source Softw.* **4**, 1686. (10.21105/joss.01686)

[34] Sjoberg D. 2022 *hablar: Non-astonishing results in R. R package version 0.3.0*. See https://CRAN.R-project.org/package=hablar.

[35] Firke S. 2021 *janitor: Simple tools for examining and cleaning dirty data. R package version 2.1.0*. See https://CRAN.R-project.org/package=janitor.

[36] Brooks ME, Kristensen K, van Benthem KJ, Magnusson A, Berg CW, Nielsen A, Skaug HJ, Machler M, Bolker BM. 2017 glmmTMB balances speed and flexibility among packages for zero-inflated generalized linear mixed modeling. R J. **9**, 378-400. (10.32614/RJ-2017-066)

[37] Hartig F. 2022 *DHARMa: residual diagnostics for hierarchical (multi-level/mixed) regression models. R package version 0.4.5.* See https://CRAN.R-project.org/package=DHARMa.

[38] Lenth R. 2022 *emmeans: Estimated marginal means, aka least-squares means. R package version 1.7.2.* See https://CRAN.R-project.org/package=emmeans.

[39] Lüdecke D. 2018 ggeffects: Tidy data frames of marginal effects from regression models. *J. Open Source Softw.* **3**, 772. (10.21105/joss.00772)

[40] Fox J, Weisberg S 2019 *An R companion to applied regression*, 3rd edn. Thousand Oaks, CA: Sage.

[41] Schielzeth H, Forstmeier W. 2009 Conclusions beyond support: overconfident estimates in mixed models. Behav. Ecol. **20**, 416-420. (10.1093/beheco/arn145) 19461866 PMC2657178

[42] Berger D, Stångberg J, Grieshop K, Martinossi-Allibert I, Arnqvist G. 2017 Temperature effects on life-history trade-offs, germline maintenance and mutation rate under simulated climate warming. Proc. R. Soc. B **284**, 20171721. (10.1098/rspb.2017.1721) PMC5698646 29118134

[43] Maklakov AA, Immler S. 2016 The expensive germline and the evolution of ageing. Curr. Biol. **26**, R577-R586. (10.1016/j.cub.2016.04.012) 27404253

[44] Smith CC, Fretwell SD. 1974 The optimal balance between size and number of offspring. Am. Nat. **108**, 499-506. (10.1086/282929)

[45] Guisande C, Sánchez J, Maneiro I, Miranda A. 1996 Trade-off between offspring number and offspring size in the marine copepod *Euterpina acutifrons* at different food concentrations. Mar. Ecol. Prog. Ser. **143**, 37-44. (10.3354/meps143037)

[46] Smith HG, Kallander H, Nilsson J-A. 1989 The trade-off between offspring number and quality in the great tit *Parus major*. J. Anim. Ecol. **58**, 383-401. (10.2307/4837)

[47] Monaghan P, Metcalfe NB. 2019 The deteriorating soma and the indispensable germline: gamete senescence and offspring fitness. Proc. R. Soc. B **286**, 20192187. (10.1098/rspb.2019.2187) PMC6939927 31847776

[48] Chen H, Jolly C, Bublys K, Marcu D, Immler S. 2020 Trade-off between somatic and germline repair in a vertebrate supports the expensive germ line hypothesis. Proc. Natl Acad. Sci. USA **117**, 8973-8979. (10.1073/pnas.1918205117) 32245815 PMC7183174

[49] Camilleri T-L, Piper MDW, Robker RL, Dowling DK. 2022 Sex-specific transgenerational effects of diet on offspring life history and physiology. *bioRxiv*, 2022.05.23.492998. (10.1101/2022.05.23.492998)

[50] Vuarin P, Bouchard A, Lesobre L, Levêque G, Chalah T, Jalme MS, Lacroix F, Hingrat Y, Sorci G. 2019 Post-copulatory sexual selection allows females to alleviate the fitness costs incurred when mating with senescing males. Proc. R. Soc. B **286**, 20191675. (10.1098/rspb.2019.1675) PMC6834038 31640511

[51] Gillespie SR, Tudor MS, Moore AJ, Miller CW. 2014 Sexual selection is influenced by both developmental and adult environments. Evolution **68**, 3421-3432. (10.1111/evo.12526) 25226860

[52] Galluccio E, Lymbery RA, Wilson A, Evans JP. 2022 Personality, sperm traits and a test for their combined dependence on male condition in guppies. R. Soc. Open Sci. **9**, 220269. (10.1098/rsos.220269) 35706668 PMC9156929

[53] Lymbery RA, Alvaro BJ, Evans JP. 2022 Does diet influence ejaculate expenditure under experimentally altered risk of sperm competition in guppies? Anim. Behav. **194**, 161-168. (10.1016/j.anbehav.2022.09.018)

[54] Devigili A, Kelley JL, Pilastro A, Evans JP. 2013 Expression of pre- and postcopulatory traits under different dietary conditions in guppies. Behav. Ecol. **24**, 740-749. (10.1093/beheco/ars204)

[55] Gasparini C, Devigili A, Dosselli R, Pilastro A. 2013 Pattern of inbreeding depression, condition dependence, and additive genetic variance in Trinidadian guppy ejaculate traits. Ecol. Evol. **3**, 4940-4953. (10.1002/ece3.870) 24455127 PMC3892359

[56] Lee W-S, Monaghan P, Metcalfe NB. 2016 Perturbations in growth trajectory due to early diet affect age-related deterioration in performance. Funct. Ecol. **30**, 625-635. (10.1111/1365-2435.12538) 27610000 PMC4994260

[57] Arendt J, Wilson DS, Stark E. 2001 Scale strength as a cost of rapid growth in sunfish. Oikos **93**, 95-100. (10.1034/j.1600-0706.2001.930110.x)

[58] Ivimey-Cook ER, Murray DS, de Coriolis J-C, Edden N, Immler S, Maklakov AA. 2023 Data from: Fasting increases investment in soma upon refeeding at the cost of gamete quality in zebrafish. Dryad Digital Repository. (10.5061/dryad.2280gb5w1) PMC10154932 37132242

[59] Maklakov AA. 2023 Fasting increases investment in soma upon refeeding at the cost of gamete quality in zebrafish. Figshare. (10.6084/m9.figshare.c.6486257)

