# Correction to: ‘Fasting increases investment in soma upon refeeding at the cost of gamete quality in zebrafish’ (2023) by Ivimey-Cook *et al.*

**DOI:** 10.1098/rspb.2023.0872

**Published:** 2023-05-10

**Authors:** Edward R. Ivimey-Cook, David S. Murray, Jean-Charles de Coriolis, Nathan Edden, Simone Immler, Alexei A. Maklakov

*Proc. R. Soc. B* **290**, 20221556 (Published Online 12 April 2023). (doi:10.1098/rspb.2022.1556)

There was a mistake in the numbering of the supplementary tables. This has now been corrected.

From:

‘(average reproduction EMM fed females: 92.7, fasted females: 105.0, contrast = 0.93, *p* = 0.566; electronic supplementary material, Table S8) resulting in a comparable total number of offspring between fed and fasted treatments (although we note the large increase in total number of offspring for females as they transition from fasting to refeeding; Total offspring: fed = 240, fasted = 281, estimate = 0.856, *p* = 0.509; electronic supplementary material, Table S9).’

To:

‘(average reproduction EMM fed females: 92.7, fasted females: 105.0, contrast = 0.93, *p* = 0.566; electronic supplementary material, table S8a), resulting in a comparable total number of offspring between fed and fasted treatments (although we note the large increase in total number of offspring for females as they transition from fasting to refeeding; total offspring: fed = 240, fasted = 281, estimate = 0.856, *p* = 0.509; electronic supplementary material, table S8b).’

